# Reviewed article: Tiroli CF, Scholze AR, Magnabosco GT, Barreto MFC, Bolorino N, Zandomenighi RC, Pieri FM. Risk factors associated with hepatitis C virus infection: population-based cross-sectional study, Paraná, 2007-2022. Epidemiol Serv Saude. 2026:35;e20250196

**DOI:** 10.1590/S2237-96222026v35e20250196.a

**Published:** 2026-04-10

**Authors:** Carolina Müller Ferreira

**Affiliations:** 1 IQVIA, São Paulo, SP, Brazil IQVIA São Paulo SP Brazil

## Peer review A

## Questionnaire

Does the manuscript contain new and significant information to justify publication? Yes

Does the Abstract (Summary) clearly and accurately describe the content of the article? Yes

Is the problem significant and concisely stated? Yes

Are the methods described comprehensively? No

Are the interpretations and conclusions justified by the results? Yes

Is adequate reference made to other work in the field? Yes

## Manuscript Structure

Length of article is: Adequate

Number of tables is: Adequate

Number of figures is: Adequate

Please state any conflict(s) of interest that you have in relation to the review of this paper. None

Interest: Good

Quality: Average

Originality: Good

Overall: Good

Recommendation: Major Revision

## Comments

A fim de agilizarmos o processo de submissão de seu manuscrito, repasso alguns ajustes a serem feitos para adequação do manuscrito aos padrões adotados pela RESS, caso o manuscrito avance para a etapa seguinte. Ressaltamos não serem necessários ajustes para o que já estiver conforme os padrões.

Referente ao conteúdo, estrutura e padronização, são necessários alguns ajustes conforme descrito abaixo:

- Resumo (Objetivo): A pergunta de pesquisa precisa ser mais bem definida considerando qual população se trata (adultos, crianças ou ambos ou há alguma faixa etária específica ou em toda a população brasileira independente da faixa etária). Considere usar a estrutura população, intervenção/exposição, comparador e desfecho na definição da pergunta de pesquisa para inserção no objetivo (PICO).

-Resumo (Conclusão):Recomenda-se que responda ao objetivo/perguntas da pesquisa propostas e sejam baseadas nos resultados. Sugiro que avalie se o objetivo engloba as característica demográficas em geral e inclua esse ponto na conclusão ou reavalie se as características todas ou alguma especifica deveria ser avaliada como objetivo da pesquisa e contemplada na conclusão.

- Introdução: A conexão entre os parágrafos deve se dar pela linearidade do texto, no qual as ideias evoluem sequencialmente. Conjunções de início de frase que pretendem trazer essa conexão devem ser evitadas, como por exemplo, "Vale ressaltar", "Vale enfatizar", “Além de”; “No entanto,”; Igualmente etc.

- Métodos: Os métodos precisam ser descritos com mais detalhes para permitir a replicação do estudo por outros pesquisadores (Pelo que está descrito, poderia se pensar em um estudo transversal que fez uso em um único momento de dados de notificação, porém também poderia ser uma coorte retrospectiva caso houve seguimento por um tempo no passado. Como não está bem detalhado, a interpretação pode ser dúbia. Sugiro explicitar melhor como foi feito, analisado, coletado e se houve algum seguimento para que o método esteja adequado e claro).

A descrição dos métodos precisa estar concordante com o guia de redação desse tipo de estudo (Transversal = Strobe). A relação completa dos guias encontra-se no site da Enhancing the QUAlity and Transparency Of health Research (EQUATOR), disponível em: http://www.equator-network.org/reporting-guidelines.

A análise estatística recomendo que seja avaliado se a razão de prevalência (típica desse tipo de desenho) não seria mais recomendada. Por se tratar de uma relação entre o desfecho e a exposição, entendo que Odds Ratio pode ter sido utilizada como a melhor forma estatística de representação dos dados, porém sugiro explicar um pouco mais as razões na metodologia. O OR pode superestimar associações em estudos transversais, então em alguns estudos recomenda-se que caso a melhor apresentação seja em OR, também apresente o RP. Sugiro que o autor avalie se cabe essa avaliação no estudo e se não, sugiro mais robustez no método ou contemplar isso na limitação do estudo.

- Discussão: A conexão entre os parágrafos deve se dar pela linearidade do texto, no qual as ideias evoluem sequencialmente. Conjunções de início de frase que pretendem trazer essa conexão devem ser evitadas, como por exemplo, “Além de”; “No entanto,”; Igualmente etc.

Recomenda-se que seja o último parágrafo da discussão e que responda ao objetivo/perguntas da pesquisa propostas e sejam baseadas nos resultados.

- Disponibilidade de dados: A disponibilidade dos dados refere-se aos dados brutos utilizados na análise descrita e abordada nesse trabalho, de modo que se possa replicar ou continuar esse estudo. Não se refere a fonte dos dados, como o banco de dados total da Secretaria de Saúde do Paraná. Reveja se será disponibilizado e caso não, sugiro mudança do texto.

-Referências: Recomenda-se citar pesquisas científicas relevantes (metodologicamente bem conduzidas, que foram avaliadas na íntegra pelos autores), atualizadas (até 5 anos), e acessíveis (artigos publicados em periódicos indexados, evitar sites e relatórios que podem se tornar indisponíveis).

Assegure-se de que as citações estejam de acordo com o estilo da revista."Devem seguir o formato Vancouver (sem doi no final):

[ARTIGO] Autor(es). Título. Nome abreviado do periódico. Ano;Volume(Número):Página. (com a página final abreviada [ex.: 123-5] ou página eletrônica [e-page]).

- Tabelas: Títulos de tabelas devem ser claros, informativos e apresentar o conteúdo da tabela ou figura. Informar o local, ano(s) e total de participantes incluídos na ilustração. Separar termos por vírgula e não incluir ponto no final dos títulos. Títulos devem ser autossuficientes para a ilustração, dispensando consultar o texto. Siglas essenciais para compreensão da ilustração devem constar preferencialmente no título, conforme exemplo: “Tabela 3. Razões de prevalências (RP) brutas e ajustadas e intervalos de confiança de 95% (IC95%) do [desfecho] pelas variáveis do estudo. Local, ano (n=xx)”.Não incluir detalhes metodológicos.

